# Obesity and Chosen Non-Communicable Diseases in PURE Poland Cohort Study

**DOI:** 10.3390/ijerph18052701

**Published:** 2021-03-08

**Authors:** Katarzyna Zatońska, Piotr Psikus, Alicja Basiak-Rasała, Zuzanna Stępnicka, Dagmara Gaweł-Dąbrowska, Maria Wołyniec, Julia Gibka, Andrzej Szuba, Katarzyna Połtyn-Zaradna

**Affiliations:** 1 Department of Social Medicine, Wroclaw Medical University, 50-345 Wrocław, Poland; Katarzyna.zatonska@umed.wroc.pl (K.Z.); zuzanna.stepnicka@gmail.com (Z.S.); dagmara.gawel-dabrowska@umed.wroc.pl (D.G.-D.); maria.wolyniec@umed.wroc.pl (M.W.); katarzyna.poltyn-zaradna@umed.wroc.pl (K.P.-Z.); 2 Calisia University, 62-800 Kalisz, Poland; burmistrz@um.kepno.pl; 3 Department of Health Sciences, Calisia University, 62-800 Kalisz, Poland; wydzial.medyczny@pwsz.kalisz.pl; 4 Department of Angiology, Hypertension and Diabetology, Wroclaw Medical University, 50-556 Wroclaw, Poland; andrzej.szuba@umed.wroc.pl

**Keywords:** PURE study, noncommunicable diseases, urban, rural, obesity

## Abstract

Introduction: Obesity has been associated with a higher risk of morbidity, disability, and death. The objective of this study was to assess the prevalence of obesity and chosen non-communicable diseases (NCDs) in the PURE Poland cohort study. Material and Methods: The study covers a group of 2035 people (1281 women and 754 men), who live in urban and rural areas of Lower Silesian voivodeship. The baseline study was conducted between 2007–2010. The data on demographic status and history of diseases were collected using questionnaires. The anthropometric parameters, blood pressure, blood lipids, and glucose level were measured. Results: Normal body weight was observed in 28.1% of participants, whereas overweight and obesity were observed in 40.1% and 31.1% of participants, respectively. Moreover, there was a significant difference in the body weight between genders. Prevalence of obesity was similar in men and women (31.0% and 31.1%, respectively). Obesity was more prevalent in rural vs. urban residents (38.5% and 26.0%, respectively). In a logistic regression analysis, the odds for obesity was two-fold higher in participants aged >64 years and rural inhabitants (OR 1.91; 95% CI 1.36–2.70; OR 1.79; 95% CI 1.48–2.16, respectively). Participants with obesity had 2.5-fold higher odds for diabetes and hypertension and two-fold higher odds for CHD in comparison with non-obese individuals (OR 2.74; 95% CI 2.01–3.73, OR 2.54; 95% CI 2.03–3.17, OR 1.88; 95% CI 1.26–2.80, respectively). Conclusions: Taken together, the prevalence of obesity was associated with particular socio-demographic factors (age, place of residence, and level of education) as well as diabetes, hypertension, and coronary heart disease.

## 1. Introduction

Obesity is one of the greatest public health challenges of the 21st century in developed and developing countries. In 1997, the World Health Organization (WHO) recognized obesity as a global health problem. In 2016, more than 1.9 billion adults were overweight worldwide. Among them, more than 650 million were obese [1]. Furthermore, according to the study of NCD Risk Factor Collaboration, between 1975 and 2014, the mean body mass index (BMI) increased across both female and male population. The study included 9.2 million people from 200 countries. The mean BMI for women increased from 22.1 kg/m^2^ to 24.4 kg/m^2^, while the mean BMI for men increased from 21.7 to 24.2 kg/m^2^. Authors concluded that if the current trend of the increase in obesity prevalence continues, the global prevalence of severe obesity will surpass the prevalence of underweight by 2025, especially in women [2].

It was estimated that in 2008, in Europe, over half of men and women were overweight, while about 23% of women and 20% of men were obese. The steady increase of excessive body weight prevalence was observed in the whole EU [3,4,5]. According to the latest estimates, overweight occurred in 30–70% of adults and obesity in 10–30% of adults in the European Union (EU) [6]. In Poland, according to the latest European Health Interview Survey (EHIS) conducted in 2014, within the population of 15 years old and above, over 62% of men and almost 46% of women were overweight, whereas 18% of men and 15.6% of women were obese [7,8]. A cross-sectional analysis of the first (2003–2005) and the second (2013–2014) edition of the Polish National Multicenter Health Survey (WOBASZ) revealed significant increase in obesity prevalence during the decade, especially in men [9].

The excessive body weight is a serious health problem, and it is a risk factor for many non-communicable diseases (NCD). Obesity is associated with a higher risk of morbidity, disability, and death. Its level of impact, as a risk for mortality, is similar to that of smoking [10]. Major risk factors for mortality are increased cardiovascular incidents, such as coronary heart disease, hypertension, stroke, type 2 diabetes, and some types of cancer, e.g., breast cancer in postmenopausal women, endometrial cancer, and colon and kidney cancer [11,12,13,14,15]. According to Fontaine et al. [16], obesity with a BMI of over 45 kg/m^2^ during early adulthood (a group of 20- to 30- year-olds) can reduce life expectancy by 13 years for men, and by eight years for women.

Overweight and obesity are relevant public health problems. It was estimated that the cost of health care for people who are overweight and obese is significantly higher than those with normal weight. Moreover, in 2014, the global economic burden of obesity contributed to the global gross domestic product (GDP) by 2.8% [17]. The cost of treating obesity and its complications in Poland absorbs 21% of the budget allocated to health [18]. That amounts to nearly 3 billion zloty, based on calculations carried out in Lubelskie Voivodeship [19]. Furthermore, diseases associated with overweight and obesity are likely to be responsible for 25% of hospital admissions (which equals approximately 1,500,000 hospital admissions across the country) according to the nationwide Polish surveys of patients in hospitals [19].

The objective of this study was to assess the prevalence of obesity and chosen non-communicable diseases (NCDs) in relation to sociodemographic factors and the association between obesity and NCDs in the PURE Poland cohort study. The PURE Poland study is one of the few large, cohort studies currently conducted in Poland. Recruitment of both urban and rural population into the study cohort is a unique approach addressing health inequalities.

## 2. Materials and Methods

The Prospective Urban and Rural Epidemiology Study (PURE) is based on data from 21 countries with different status of economic development. Poland, at the beginning of the research, was one of the seven upper-middle income countries enrolled in the study [20]. All participants were tested in accordance with the PURE study protocol [21]. This paper presents the results of the PURE Poland study—baseline. The data were collected between 2007 and 2010. Among 2035 participants there were 1281 women and 754 men who inhabited urban and rural areas of Lower Silesia. They were divided into three age groups (<45, 45–64, and >64).

Collection of data concerning individuals was obtained at two levels [21]. First—Family/Household level included demographic information, i.e., number of family/household members, number of children, education, etc.; epidemiological information, i.e., morbidities, usage of tobacco products, etc.; and other important determinants, i.e., access to water, sanitation, household amenities, etc. All this information was collected using Family Census Questionnaire. Second—Adult participants’ level, which was collected based on an Adult Questionnaire. This included data on nine INTERHEART risk factors (lipids, smoking, hypertension, diabetes, abdominal obesity, psychosocial factors (stress and symptoms of depression), consumption of fruits and vegetables, consumption of alcohol, and regular physical activity), anthropometric measures, blood pressure, spirometry, fasting blood sample, semi-quantitative food frequency questionnaires (FFQs), and physical activity (IPAQ).

The body mass of the patients was measured using Tanita Ironman Body Composition Monitor Model BC-554 with accuracy of 0.1 kg. The BMI was calculated as weight (kg) divided by height (m) squared. Subjects were classified into four BMI categories, according to the WHO guidelines: underweight (BMI < 18.5 kg/m^2^), normal weight (BMI 18.5–24.9 kg/m^2^), overweight (BMI 25.0–29.9 kg/m^2^), and obese (BMI ≥ 30.0 kg/m^2^). Diabetes was ascertained on the basis of (1) self-reported diabetes and/or (2) self-reported anti-diabetic medication and/or (3) fasting blood glucose measurement ≥126 mg/dL [22]. Hypertension was ascertained on the basis of (1) self-reported hypertension and/or (2) self-reported anti-hypertensive medication and/or (3) an average of two blood pressure measurements ≥140/90 mmHg, as previously described [23]. The occurrence of coronary heart disease (CHD) and stroke was self-reported by the participants. Due to lack of data regarding aforementioned variables, diabetes was assessed in a total of 1663 participants, hypertension in 2019 participants, and CHD and stroke in 2029 participants.

The aggregated information on the prevalence of obesity and NCD was presented using basic statistical parameters. The prevalence of obesity and NCD was verified with respect to epidemiological variables known to be its significant determinants and health related states correlated to obesity and/or NCD. To assess the statistical significance of the differences observed in the distribution of obesity and NCD, the chi-square test was used. The association between sociodemographic/anthropometric factors with NCDs was assessed with the use of adjusted logistic regression models after adjusting for age and/or gender. The strength of the association was measured by the odds ratio (OR) with 95% confidence intervals. For all differences, the level of the statistical significance was *p* ≤ 0.05. Statistical analysis was conducted using the software Statistica 13.1 PL (StatSoft Inc., Palo Alto, CA, USA).

## 3. Results

A baseline characteristics of the study population in the view of body mass index categories is presented in Table 1. There were 28.1% of participants with normal body weight, while 40.1% and 31.1% of participants were overweight and obese, respectively. Moreover, there were statistically significant differences in the distribution of body weight according to sex, age, marital status, level of education, and place of residence. The occurrence of chosen NCDs (stroke, CHD, hypertension, diabetes) was also differentiated by the BMI.

The normal body weight was more prevalent in women than in men (32.3% and 20.8%, respectively). We observed similar prevalence of obesity in women and men (31.1% vs. 31.0%, respectively). Furthermore, the prevalence of overweight and obesity significantly increased in participants >44 years of age. Obesity was observed in 19.8% of participants aged 30–44 years, 32.7% in participants aged 45–64 years, and 36.7% in participants aged >64 years, respectively. Moreover, the prevalence of obesity decreased stepwise with higher level of education (45.2% of obese individuals in participants with primary education vs. 22.7% in participants with higher education, respectively). On the contrary, the prevalence of overweight increased with increasing level of education. Additionally, participants who were separated/divorced were more likely to be obese than married or never married/single participants (35.2% vs. 30.5%, 26.7%, respectively), whereas married participants were more likely to be overweight than separated or never married participants (41.2% vs. 36.0%, 38.4%, respectively). Finally, normal body weight was more prevalent in urban residents compared with those living in rural areas (30.2% and 25.0%, respectively). Obesity was much more prevalent in rural inhabitants than in urban (38.5% vs. 26.0%, respectively), whereas overweight was more prevalent in urban inhabitants (43.1% vs. 35.8%).

In a performed logistic regression analysis, the factors that were significantly associated with increased odds for obesity included: rural place of residence, age, and education level (Table 2). In an adjusted model, the odds for obesity were over 1.5-fold higher among rural residents compared with urban residents (odds ratio [OR] = 1.79, 95% CI = 1.48–2.16) (Table 2). The was no significant difference in the occurrence of obesity between men and women. The odds for obesity increased with increasing age of participants. The highest odds for obesity were observed in the age group >64 years of age [OR 1.91, 95% CI 1.36–2.7]. It was almost two-fold higher than in the group of 30- to 44-year-olds. Furthermore, with increasing level of education, the odds of obesity significantly decreased ([OR] = 0.42, 95% CI 0.34–0.56 in participants with higher education).

**Figure 1:**
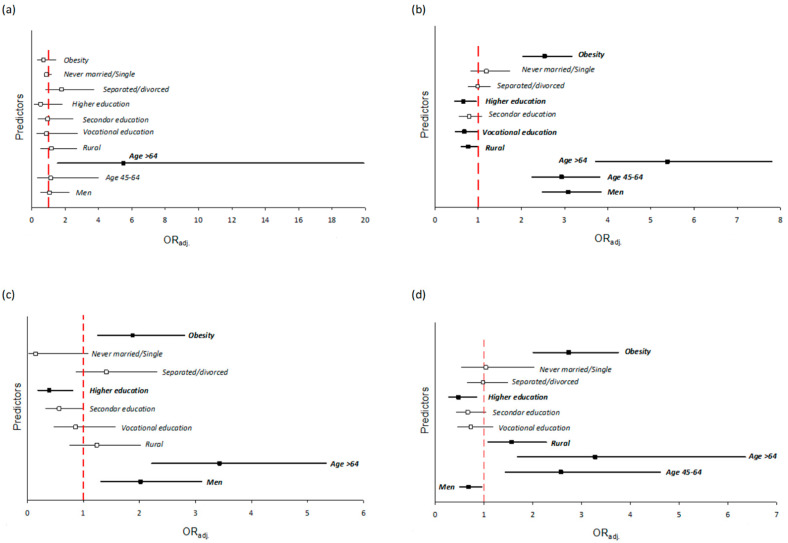
The odds ratios (with 95% CI) of the factors associated with occurrence of stroke (**a**), hypertension (**b**), CHD (**c**), and diabetes (**d**) among the study population (the odds ratios were adjusted to sex and/or age, as indicated previously in Table 2).

Finally, the odds ratios of the factors associated with occurrence of stroke, hypertension, CHD, and diabetes among the study population were presented in Figure 1.

The odds for stroke were significantly associated with increasing age (Figure 1a). Participants aged >64 years had 5.5-fold higher odds for stroke than 30- to 44-year-olds (OR 5.49; 95% CI 1.51–19.9). The odds for stroke was not associated with obesity, gender, place of residence, education, or marital status.

Factors independently differentiating the odds for hypertension included gender, age, obesity, place of residence, and level of education (Figure 1b). The increasing age was the factor associated with the highest odds for hypertension. Participants aged > 64 years had over five-fold higher odds for hypertension, and 45- to 64-year-olds had almost three-fold higher chance, than 30- to 44-year-olds (OR 5.38, 95% CI 3.71–7.80, OR 2.93, 95% CI 2.25–3.82, respectively). Men had three-fold higher odds for hypertension than women (OR 3.09; 95% CI 2.48–3.84). Obesity was associated with 2.5-fold higher odds for hypertension [OR 2.54; 95% CI 2.03–3.17]. Higher and vocational education were associated with 1.5-fold lower odds for hypertension in comparison to primary education [OR 0.66; 95% CI 0.46–0.95, OR 0.68; 95% CI 0.47–0.98, respectively]. Rural inhabitants had 1.3-fold lower odds for hypertension than urban inhabitants [OR 0.77; 95% CI 0.60–0.97].

The occurrence of coronary heart disease was independently associated with factors like gender, age, level of education, and obesity (Figure 1c). The odds for CHD were over three-fold higher in participants aged >64 years than in younger participants [OR 3.43; 95% CI 2.22–5.33]. Men had two-fold higher odds for CHD than women [OR 2.02; 95% CI 1.31–3.11]. Obese individuals had almost two-fold higher odds for CHD [OR 1.88; 95% CI 1.26–2.80]. Higher level of education was associated with 2.5-fold lower risk of CHD than primary education [OR 0.39; 95% CI 0.19–0.81]. The odds for occurrence of CHD was not differentiated by the place of residence.

Factors independently associated with increased odds for diabetes included age, obesity, place of residence, gender, and the level of education (Figure 1d). The odds for diabetes increased with increasing age of the population. Participants aged >64 years had three-fold higher odds for diabetes than 30- to 44-year-olds [OR 3.28; 95% CI 169–6.36]. Participants with obesity had over 2.5-fold higher odds for diabetes than non-obese individuals [OR 2.74; 95% CI 2.01–3.73]. Place of residence also differentiated the risk of diabetes. Participants living in rural areas had 1.5-fold higher odds for diabetes than participants living in urban areas [OR 1.57; 95% CI 1.08–2.28].

## 4. Discussion

In Poland, over the last ten years, a consecutive increase in the prevalence of overweight and obesity was observed for both genders. In the representative Polish National Multicenter Health Survey (WOBASZ) conducted in 2003–2005 among 20- to 74-year-olds, overweight was diagnosed in 61% of men and in 50% of women, and obesity in 21% and 22%, respectively [24]. There was a significant increase in obesity prevalence during the decade, especially in men. In the second edition, WOBASZ II conducted in 2013–2014, obesity was observed in 24.4% of men and 25.0% of women [9]. In the Central Statistical Office report on the health state of the Polish population aged 15 and above published in 2015, 41.1% of men and 30.1% of women were overweight, whereas 18.1% of men and 15.6% of women were obese [7]. The average BMI for men in 2008 increased by 1.6 kg/m^2^ compared with the year 1980 and was 26.7 kg/m^2^ [25]. In women, the average BMI did not change between 1980 and 2008, and it was 25.9 kg/m^2^ [25]. In a Polish-Norwegian Study (PONS) on 3854 inhabitants of Świętokrzyskie Voivodeship aged 45–64, the average BMI in men was 28.5 kg/m^2^ and in women—28.2 kg/m^2^. That summed up to 52% of men and 42% of women being overweight, whereas 35% of men and women being obese [26].

In our study, the prevalence of obesity was associated with particular socio-demographic factors. It was higher among people in older age groups and lower education levels. These results were consistent with previously published literature on the association of obesity with respect to age and educational level; for instance, the PONS study [26], the Vanio et al. study [27], and others [9,28,29]. Furthermore, based on the population of the NHANES study [30], obesity was much more prevalent among inhabitants of rural than urban areas, which is consistent with our findings as well. In a longitudinal cohort study conducted by Patterson et al. [31], a greater cumulative exposure to rurality during early adulthood was associated with higher risk of obesity in middle-aged adults. Similarly, in a study by Jokela et al. [32], rural residence was more associated with higher BMI than urban residence. Our results suggest that individuals with lower educational level as well as older people are statistically more likely to be obese. On the other hand, we observed higher prevalence of overweight in participants with higher level of education and participants living in urban areas.

According to the WHO, the risk of type 2 diabetes, insulin resistance, or dyslipidaemia is over three-fold higher in obese people than in those with normal body weight. The risk of the coronary heart disease and hypertension is 2–3-fold higher in obese people, while the risk of cancer is 1–2-fold higher in obese people compared with those with normal body weight [33,34]. In a study by Mongraw-Chaffin et al. [35], the increment in the BMI was associated significantly with the risk of coronary heart disease in both genders. Authors observed that in comparison to participants with normal body weight, the HR of coronary heart disease in participants with obesity was 1.6 [1.42–1.82] in women and 1.60 [1.43–1.79] in men [35]. We observed that obesity was independently associated with increased odds for diabetes, hypertension, and CHD. Similar results were obtained by Field et al. [36], who analyzed data on 10-year follow-up of Nurses’ Health Study. Authors found that the incidence of diabetes, hypertension, heart disease, and stroke (only for men) elevated with increasing overweight in both men and women. Furthermore, results obtained from both NATPOL 2011 study in Poland and Polish-Norwegian Study (PONS) also suggest much higher prevalence of diabetes among obese individuals in comparison with individuals with normal body weight [26,37]. Although we cannot establish the causality, since both BMI and occurrence of NCDs were reported at the baseline study, our findings suggest a significant association between occurrence of those conditions.

The social burden of obesity is clear when comparing obesity related healthcare (44% higher) and patients with a regular body mass [38]. This is due to the obesity being a risk factor for morbidity. The obesity-related comorbidities are hypertension, reduced insulin sensitivity, diabetes mellitus, some types of cancer, and various heart diseases. Obesity is related to increased blood pressure and increased prevalence of CHD. Increase in body mass by 10 kg was associated with the increase of systolic blood pressure by 3 mmHg. Consequently, this further results in 12% increase of CHD risk [39]. The AHA (American Heart Association) recommended several important areas of further research on CVD and obesity. One of them focuses on “policy research on the impact of overweight/obesity on the future health care in people with or without CVD”.

Considering the health-related and economic cost of obesity, this issue should be addressed by health policy makers. Policies aiming to decrease the social burden of obesity and reduce the number of obesity-related comorbidities can be found in series of important documents, i.e., the Polish National Healthcare Program, Polish National Civilization Diseases Prevention Program, and Global Strategy on Nutrition, Physical Activity, and Health signed at the 57th World Health Forum in May 2004, European Charter on Counteracting Obesity, and others [38]. Actions are taken in two key areas: increasing the knowledge and modification of eating habits and increasing physical activity for better energy balance. The prevalence of obesity in our study was high. Considering the high association between obesity and non-communicable diseases, our observations suggest that our study population is at the high risk of further metabolic disorders. People with overweight and obesity should be the group of special focus when planning prophylactic programs.

There are some limitations to consider. The PURE study is a longitudinal prospective cohort study, which has been continued in follow-ups every three years. In the present study, we analyzed data from baseline only. The data was collected between 2007 and 2010. The study group was selected with the use of a snowball sampling method, which can introduce a possible bias. Either in comparison to the overall population of Poland or the population of Lower Silesia, our study population is characterized by the overrepresentation of women and participants with higher education. Both the occurrence of NCDs and sociodemographic and anthropometric factors were reported at the baseline, which impede the possibility to draw conclusions about the causality of observed associations. Additionally, the number of stroke cases at the baseline was limited, and this fact potentially decreased the accuracy of statistical analysis in case of this condition. We plan to address this issue in future research of prospective data of our cohort.

## 5. Conclusions

The prevalence of obesity observed in the PURE Poland study baseline population was associated with particular socio-demographic factors. The increased odds for obesity were associated with older age and rural place of residence. The level of education was inversely associated with occurrence of obesity. We observed that obesity as well as increasing age were independently associated with increased odds for diabetes, hypertension, and CHD. Observed associations can be helpful to develop more adequate health promotion programs.

## Figures and Tables

**Table 1:** A baseline characteristics of the study population in the view of body mass index categories.

Variables	*N*	BMI (kg/m^2^)				*p*-Value *
		<18.5 *N* = 15	18.5–24.9 *N* = 571	25.0–29.9 *N* = 817	≥30.0 *N* = 632	
Total	2035	0.7% (15)	28.1% (571)	40.1% (817)	31.1% (632)	
Sex:						<0.001
Men	754	0.4% (3)	20.8% (157)	47.8% (360)	31.0% (234)	
Women	1281	0.9% (12)	32.3% (414)	35.7% (457)	31.1% (398)	
Place of residence:						<0.001
Urban	1210	0.7% (9)	30.2% (365)	43.1% (522)	26.0% (314)	
Rural	825	0.7% (6)	25.0% (206)	35.8% (295)	38.5% (318)	
Age, years:						<0.001
30–44	358	1.4% (5)	43.6% (156)	35.2% (126)	19.8% (71)	
45–64	1350	0.6% (8)	25.9% (350)	40.8% (551)	32.7% (441)	
>64	327	0.6% (2)	19.9% (65)	42.8% (140)	36.7% (120)	
Level of education:						<0.001
Primary	301	0.7% (2)	18.2% (55)	35.9% (108)	45.2% (136)	
Vocational	324	1.2% (4)	25.9% (84)	37.1% (120)	35.8% (116)	
Secondary	796	0.6% (5)	28.6% (228)	40.5% (322)	30.3% (241)	
Higher	603	0.7% (4)	33.7% (203)	42.9% (259)	22.7% (137)	
Lack of information	11	0.0% (0)	9.1% (1)	72.7% (8)	18.2% (2)	
Marital status:						0.016
Married/living together	1508	0.7% (10)	27.6% (417)	41.2% (621)	30.5% (460)	
Separated/divorced	375	0.3% (1)	28.5% (107)	36.0% (135)	35.2% (132)	
Never married/single	146	2.7% (4)	32.2% (47)	38.4% (56)	26.7% (39)	
Lack of information	6	0.0% (0)	0.0% (0)	83.3% (5)	16.6% (1)	
Stroke	38	0.0% (0)	23.7% (9)	47.4% (18)	28.9% (11)	0.767
CHD	112	0.0% (0)	16.1% (18)	34.8% (39)	49.1% (55)	<0.001
Hypertension	1217	0.5% (6)	19.2% (234)	41.7% (507)	38.6% (470)	<0.001
Diabetes	200	0.0% (0)	12.5% (25)	30.0% (60)	57.5% (115)	<0.001

* Chi-square test.; BMI—Body Mass Index; CHD—Coronary Heart Disease.

**Table 2:** The adjusted odds ratio (OR) of the occurrence of obesity in the study population.

Variable	Obesity				*p*-Value	OR_adj._ (95% CI)
	Yes *N* = 632		No *N* = 1403			
	*n*	%	*n*	%		
Sex:					0.974	
Women	398	63.0	883	62.9		1.00 (ref.)
Men	234	37.0	520	37.1		0.99 (0.81–1.20) ^a^
Age group:					<0.001	
30–44	71	11.2	287	20.5		1.00 (ref.)
45–64	441	69.8	909	64.8		1.76 (1.42–2.17) ^b^
>64	120	19.0	207	14.8		1.91 (1.36–2.70) ^b^
Place of residence:					<0.001	
Urban	314	49.7	896	63.9		1.00 (ref.)
Rural	318	50.3	507	36.1		1.79 (1.48–2.16) ^c^
Level of education:					<0.001	
Primary	136	21.6	165	11.8		1.00 (ref.)
Vocational	116	18.4	208	14.9		0.84 (0.75–0.93) ^c^
Secondary	241	38.3	555	39.8		0.69 (0.66–0.95) ^c^
Higher	137	21.7	466	33.4		0.42 (0.34–0.56) ^c^
Marital status:					0.105	
Living together	460	72.9	1048	75.0		1.00 (ref.)
Separated	132	20.9	243	17.4		0.84 (0.75–1.23) ^c^
Single	39	6.2	107	7.7		0.88 (0.79–1.19) ^c^

^a^ OR_adj._: odds ratio adjusted for age, ^b^ OR_adj._: odds ratio adjusted for sex, ^c^ OR_adj._: odds ratio adjusted for sex and age; ref.—reference value.

## Data Availability

Restrictions apply to the availability of these data. Cohort data was collected as a part of international Prospective Urban and Rural Epidemiological Study. Any requests regarding availability of data should be directed to the study supervisors.
